# Predicting the Metabolic Sites by Flavin-Containing Monooxygenase on Drug Molecules Using SVM Classification on Computed Quantum Mechanics and Circular Fingerprints Molecular Descriptors

**DOI:** 10.1371/journal.pone.0169910

**Published:** 2017-01-10

**Authors:** Chien-wei Fu, Thy-Hou Lin

**Affiliations:** Department of Pharmacy, National Taiwan University Hospital Hsin-Chu Branch, Institute of Molecular Medicine and Department of Life Science, National Tsing Hua University, HsinChu, Taiwan, ROC; Harbin Institute of Technology Shenzhen Graduate School, CHINA

## Abstract

As an important enzyme in Phase I drug metabolism, the flavin-containing monooxygenase (FMO) also metabolizes some xenobiotics with soft nucleophiles. The site of metabolism (SOM) on a molecule is the site where the metabolic reaction is exerted by an enzyme. Accurate prediction of SOMs on drug molecules will assist the search for drug leads during the optimization process. Here, some quantum mechanics features such as the condensed Fukui function and attributes from circular fingerprints (called Molprint2D) are computed and classified using the support vector machine (SVM) for predicting some potential SOMs on a series of drugs that can be metabolized by FMO enzymes. The condensed Fukui function f_A_^−^ representing the nucleophilicity of central atom A and the attributes from circular fingerprints accounting the influence of neighbors on the central atom. The total number of FMO substrates and non-substrates collected in the study is 85 and they are equally divided into the training and test sets with each carrying roughly the same number of potential SOMs. However, only N-oxidation and S-oxidation features were considered in the prediction since the available C-oxidation data was scarce. In the training process, the LibSVM package of WEKA package and the option of 10-fold cross validation are employed. The prediction performance on the test set evaluated by accuracy, Matthews correlation coefficient and area under ROC curve computed are 0.829, 0.659, and 0.877 respectively. This work reveals that the SVM model built can accurately predict the potential SOMs for drug molecules that are metabolizable by the FMO enzymes.

## Introduction

The flavin-containing monooxygenase (FMO) is a flavoprotein which carries a flavin adenine dinucleotide (FAD) and utilizes NADPH and oxygen to catalyze the metabolism of many xenobiotics such as compounds containing nitrogen, sulfur, selenium, phosphorous and other nucleophilic heteroatoms [1–4]. The family of mammalian FMO genes is comprised with five similar genes from FMO1 to FMO5 and all of them are important Phase I metabolic enzymes as being capable of metabolizing xenobiotics. FMO1 and FMO3 are the two major isoforms expressed in liver microsomes and other tissues. While FMO1 is highly expressed in fetal liver, FMO3 is predominantly found in adult human. However, FMO2 is expressed overwhelmingly in lung and fewer FMO4 and FMO5 isoforms are found in human body [3, 5]. People may suffer the so called "fish odor syndrome" when their FMOs are mutated or defected and failing to metabolize trimethylamine such as trimethylamine *N-*oxide to its oxygenated form for converting it into urine and sweat [6].

Both FMOs and cytochrome P450 (CYP450) are Phase I metabolic enzymes involving in the metabolism of xenobiotics in human. The main function of these microsomal enzymes is to add oxygen to the foreign compounds and render these foreign compounds to soluble form so that they can be excreted out of human body. Although both FMOs and CYP450 sometimes metabolize the same type of chemical compounds, the metabolic mechanisms exerted by these two enzymes are fundamentally different. While the CYP450 enzymes oxidize compounds through some electrophilic reactions and create some radical intermediates, the FMO enzymes oxidize compounds through the nucleophilic addition reactions [5, 7]. The substrates of the two enzymes are also quite different. The FMO enzymes tend to metabolize molecules with soft nucleophilic atoms such as N, S, P, and Se, whereas the CYP450 systems not only oxidize molecules with these types of atoms but also directly metabolize the C atom and few of this type is found by FMO enzymes.

Though the substrate range of FMO appear to be somewhat narrower than that of CYP450, the enzyme system still plays a crucial role in Phase I metabolism of xenobiotics [7, 8]. For examples, some therapeutic agents such as benzydamine, itopride, and arbidol are primarily metabolized by FMO but not by CYP450 enzymes [9–11]. However, some are both metabolized by FMO and CYP450 enzymes like diphenhydramine and ziprasidone [12–14]. Moreover, some metabolites of FMO enzymes are also known to be the substrates of CYP450 such as TG100435, a Src kinase inhibitor [15]. The paramount difference between FMO and CYP450 is that unlike the latter, the former is not easily induced nor readily inhibited thereby the potential adverse drug-drug interaction may be prevented for drugs predominantly metabolized by the former [7, 8]. These properties may offer advantages in drug design and discovery. By taking the FMO detoxication pathways into account in designing new drugs, more meaningful drug-like materials may be found.

The site of metabolism (SOM) in a molecule is defined as the place where the reaction of metabolism can take place. Identification of SOMs is important in the drug optimization process for searching the drug leads. The information of SOM identified may guide developers to optimize the drug structure through changing the specific sites to avoid the unwanted metabolic reactions. However, identifying SOM through experimental procedures is not an easy task since each metabolite of synthesized compound has to be isolated and then characterized through some specific techniques such as LC-MS-MS [16]. Thus, acquiring information of SOM through experimental works is a highly cost and time consuming process. Therefore, developing accurate in silico methods for predicting SOM may be worthwhile and important.

There are several in silico methods for predicting SOM for drugs that can be metabolized by CYP450 have been developed. These approaches can be roughly divided into the following two categories namely, the ligand (substrate) based and the structure (enzyme) based ones. For examples, SMARTCyp [17] is a method that using a database of activation energies computed from quantum mechanics for a variety of ligand fragments and an accessibility descriptor computed to rank all the possible SOMs. IMPACTS [18] combines the docking result given by the molecular docking program GOLD, a reactivity index with computed hydrogen bond order descriptors and the local ionization energy to refine the prediction for SOMs [19, 20]. Metasite combines the ligand and structure modeling together to predict SOM and the FMO enzymes are recruited into their package recently [21]. There are also machine learning techniques being developed for predicting SOM. MetaPrint2D [22] is a online tool that is based on training the Accelrys Metabolite Database [23] and then predicting SOM through counting occurrences of atomic fingerprints exhibiting as SOM or non-SOM in the database. Another example is RegioSelectivity (RS)-predictor [24, 25] which uses a Support Vector Machine (SVM) to predict SOM through training on 148 topological and 392 quantum mechanics atomic descriptors including contributions from neighbouring atoms.

There are other machine learning approaches using different descriptors to predict SOM such as Xenosite [26] using the Daylight fingerprint descriptors [27]. A SVM method [28] using the Kyoto Encyclopedia of Genes and Genomes (KEGG) pathway database [29] and other various descriptors has been published. FAME [30] is a broad metabolism predictor using the Random Forest model and the atomic descriptors calculated from CDK [31]. A method using the decision trees on the activation energies and solvent accessible surface area calculated from MOE [32–35] has been developed. Recently, the PASS algorithm using some 2D fingerprint descriptors [36] or the RASCAL (Random Attribute Subsampling Classification) algorithm [37] using some circular fingerprints has been employed to predict SOM by CYP450 enzymes [38, 39].

**Fig 1:**
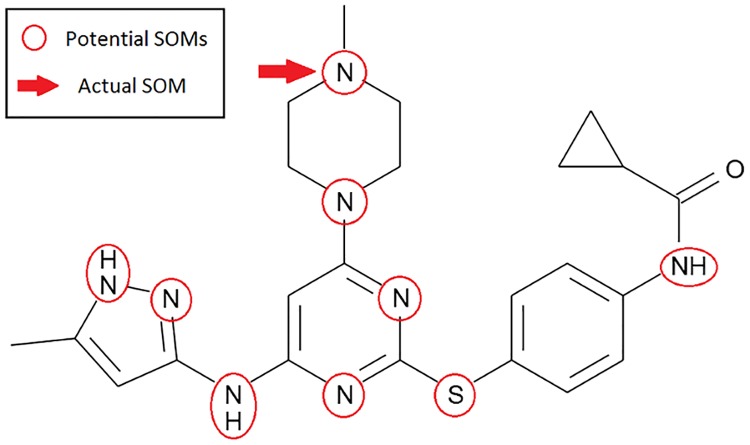
An example of FMO substrate and its SOMs. The potential SOMs identified for Tozasertib which is one of the FMO substrates studied. The potential SOMs are marked with red circles while the actual SOM is highlighted by a red arrow.

In this study, we predict SOM by FMO enzymes through using SVM on some quantum mechanics and a circular fingerprints method Molprint2D. Only the substrates-based information is used here to predict SOMs by FMO enzymes. The potential SOMs on some FMO substrates are marked first. These are usually atom N and S which account for over 90% presence in FMO metabolites. An example of a marked FMO substrate is shown in Fig 1. Next, the local reactivity of these potential SOMs is characterized through the quantum mechanics calculation. These quantum mechanics plus attributes from Molprint2D fingerprints computed are then classified by SVM to predict whether a site could be a SOM or not. The prediction accuracy of our SVM models are also validated using both internal and external data sets. While SVM has been successfully used for predicting SOMs for some substrates of CY450 enzymes, it has not been implemented for predicting SOMs for substrates of only FMO enzymes like the work presented herein.

## Materials and Methods

### Datasets

Unlike the CYP450 system, the available FMO substrates are far less. For example, only eight substrates of FMO3 in the Human Metabolome Database (HMDB) are listed [40]. However, we have collected a total of 85 compounds from literatures [11, 13, 15, 21, 41–86] for making a dataset of 228 potential SOMs. The 85 compounds collected including 73 FMO substrates and 12 non FMO substrates. These 228 instances were randomly divided into a training and a test set using the Research Randomizer serve. The number of compounds allocated in the training and the test set were respectively 42 and 43 (Table 1). Moreover, the number of SOMs and non-SOMs instances identified in each set was shown in Table 2. Only two major FMO-catalyzed reactions namely N-oxidation and S-oxidation were considered in this study since the data for C-oxidation was scarce. The actual SOMs or non-SOMs on each compound were visually defined and marked. After dividing the dataset into the training and the test sets, the number of instances counted for the former and the latter were 111 and 117, respectively.

**Table 1:** Dividing the 85 compounds collected into the training and the test sets.

Number	compound name	reaction	Potential som	som or nonsom
**1**	**(S)-nicotine**	**N OX**	**(S)-nicotine 3 N**	**nonsom**
			**(S)-nicotine 7 N**	**som**
**2**	**clozapine**	**N OX**	**clozapine 3 N**	**nonsom**
			**clozapine 4 N**	**nonsom**
			**clozapine 10 N**	**nonsom**
			**clozapine 18 N**	**som**
**3**	**cysteamine**	**S OX**	**cysteamine 3 N**	**nonsom**
			**cysteamine 4 S**	**som**
**4**	**disulfoton**	**S OX**	**disulfoton 2 S**	**nonsom**
			**disulfoton 5 S**	**nonsom**
			**disulfoton 12 S**	**som**
**…**	**…**	**…**	**…**	**…**
**↓Assigned by Research Randomizer**				
**Compound 2, 3, 9, 13, 14, 18, 22, 23, 24, 26, 27, 28, 30, 31, 32, 33, 35, 37, 39, 40, 41, 46, 47, 49, 50, 52, 54, 57, 59, 60, 61, 64, 66, 68, 70, 72, 74, 75, 77, 79, 82, 85 were assigned as the test set**				

First, the total collected compounds was arranged and numbered in order of collection time. Then, 42 out of 85 compounds were assigned as the test set by the web server Research Randomizer and the rest were treated as the training set.

**Table 2:** The total instances assigned for the training and the test sets were shown.

Dataset	Number of compounds	Substrates of FMO	Non substrates of FMO	Number of instances (Potential SOMs)	Number of actual SOMs	Number of non-SOMs
**The data for training with 10 CV**	**43**	**40**	**3**	**111**	**44**	**67**
**Test set**	**42**	**33**	**9**	**117**	**36**	**81**

### Quantum mechanics features

We employed the condensed Fukui reactivity indices as the main quantum mechanics features to represent the local reactivity of atoms within a molecule since molecules with larger values of Fukui function computed may show higher reactivity [87–92]. The Fukui function was defined as follows:

$$f\left(\vec{r}\right)={\left(\frac{\partial \rho \left(\vec{r}\right)}{\partial N}\right)}_{v}$$ (1)

Where $\rho \left(\vec{r}\right)$ was electronic density, N was number of electrons and ν was external potential exerted by the nuclei. The concept was first described by Fukui in 1952 [93] and a corresponding definition with the Density functional theory (DFT) was given in 1984 [89, 91]. However, the condensed Fukui function was restricted to an atom within a molecule rather than a point in 3D space [94–98]. The condensed Fukui function or the Fukui reactivity indices of atom A in a molecule M were defined as follows:

$${\text{f}}_{\text{A}}{}^{+}={\text{P}}_{\text{A}}\left(\text{N}+1\right)-{\text{P}}_{\text{A}}\left(\text{N}\right)$$ (2)

$${\text{f}}_{\text{A}}{}^{-}={\text{P}}_{\text{A}}\left(\text{N}\right)-{\text{P}}_{\text{A}}\left(\text{N}-1\right)$$ (3)

fA0=12[PA(N+1)−PA(N−1)] (4)

where f_A_^+^ was the electrophilicity of atom A, f_A_^−^ was the nucleophilicity of atom A, f_A_^0^ was the radical attack susceptibility of atom A, P_A_(N) was the population on atom A with N electrons, P_A_(N+1) was the population on atom A with N+1 electrons, and P_A_(N-1) was the population on atom A with N-1 electrons. While P_A_(N) was computed from the Mulliken charges, P_A_(N) was computed as atomic number of atom A—q_A_(N), where q_A_(N) was the charge on atom A with N electrons. The structures of all the 85 compounds were optimized in gas phase using the hybrid B3LYP functional and the 6-31G(d,p) basis set [99–103]. Then, the three population states with N, N+1 and N-1 electrons were calculated using the optimized structures with the same basis set. The PCM solvation model was subsequently employed to calculate the surface area for each atom. The charges, parameters of condensed Fukui function, and surface area of atoms computed were used in the training process. All the aforementioned calculations were performed using the Gaussian 09 package [104].

### Attributes from circular fingerprints

**Fig 2:**
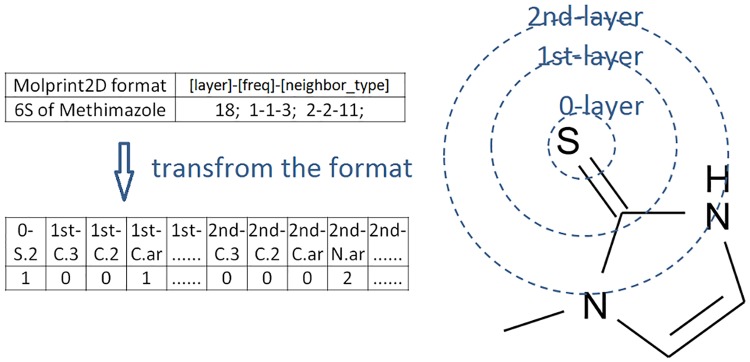
An example of attributes generated by Molprint2D and its definition. The original format of Molprint2D has been transformed by us into some numerical values for being readable by libSVM in the WEKA package.

The Fukui reactivity indices described above represented the local reactivity of a specific atom in a molecule and was insufficient to account the influence from neighbor atoms. To include the neighbor influence, we used Molprint2D [105] to evaluate the effect of neighbor atoms around each potential SOM. Molprint2D recognized the SYBYL atom type and counted the occurrence times of a neighbor atom of a particular atom type. The occurrence times counted for different neighbor atoms of different atom types were treated as the molecular descriptors. For a specific atom, all its neighbor atoms were generated iteratively by chemical bond lengths defined as follows:

[atomtype];

[1st-layer]-[frequency]-[neighbour_type]; [2nd-layer]-[frequency]-[neighbour_type]; [3rd-layer]-[frequency]-[neighbour_type]; …;

where [atomtype] was the atom type of a center atom designated by a number representing the atom type identified from all the 53 SYBYL atom types namely from C.3, C.2, C.ar,…, to Co.oh, [1st-layer]-[frequency]-[neighbour_type] represented its neighbor atom types within one bond, [2nd-layer]-[frequency]-[neighbour_type] represented its neighbor atom types within two bonds and so on. An example of attributes from Molprint2D fingerprints was depicted in Fig 2. The Molprint2D fingerprints computed for each drug molecule were converted to a series of numeric attributes for being readable by a machine learning package. Here, we restricted the scope of each Molprint2D fingerprint computed to two chemical bond lengths. Further, the Molprint2D fingerprint for each drug molecule was generated using the Open Babel package [106].

### SVM classifier

The Support Vector Machine (SVM) classifier was used to predict whether an atom within a molecule could be a SOM of FMO enzymes or not. SVM can classify complex, non linear and high dimensional data into two classes. The merit of SVMs is to classify data by mapping input vectors into a high- or infinite- dimensional space with some kernel functions and then constructing a hyperplane or set of hyperplanes to separate them into two classes with a possible maximal margin computed. The margin is defined as the distance from the separating hyperplane to the nearest training-data point. The trained model of a SVM classifier can be used to predict to which class an unknown sample is belonging. Details on the basic SVM theory can be found elsewhere [107–109]. Here, we used the free SVM program LibSVM in the WEKA package [110] to perform all the classification tasks. Much of the running parameters used were default settings except *C* and γ values. We have selected the AUC based settings for training and then the best parameters obtained after training were used for predicting for the test set. Parameter optimization was performed by the package automatically using a grid-based search procedure.

### Performance measures

There were several model validation methods such as independent data test, n-fold cross-validation, jackknife (leave-one-out) cross-validation could be used to estimate the predictability of our SVM models built [111–114]. Due to fewer features (42 features) and samples (85 compounds) used, we have employed both the 10-fold cross-validation and jackknife (leave-one-out) methods for validating our SVM models built. A web available method, the Research Randomizer was used to rationally divided our samples into a training and a test set. To avoid arbitrariness or bias, the SVM models built by the training set were validated not only by the 10-fold cross validation but also by the jackknife methods.

The prediction performance of the SVM models constructed was assessed through computing the following parameters: sensitivity (SE), specificity (SP), accuracy (ACC), and Matthews correlation coefficient (MCC) on the test set. These parameters were defined as follows:

$$\text{Sensitivity(SE)}=\frac{\text{TP}}{\text{TP }+\text{ FN}}$$ (5)

$$\text{Specificity(SP)}=\frac{\text{TN}}{\text{TN }+\text{ FP}}$$ (6)

$$\text{Accuracy(ACC)}=\frac{\text{TP }+\text{ TN}}{\text{TP }+\text{ FP }+\text{ TN }+\text{ FN}}$$ (7)

$$\text{MCC}=\frac{\left(\text{TP}\right)\left(\text{TN}\right)\;-\;\left(\text{FP}\right)\left(\text{FN}\right)}{\sqrt{\left[\text{TP }+\text{ FP}\right]\left[\text{TP }+\text{ FN}\right]\left[\text{TN }+\text{ FP}\right]\left[\text{TN }+\text{ FN}\right]}}$$ (8)

where TP, FN, TN, and FP stood for true positive, false negative, true negative and false positive, respectively. In addition to ACC and MCC, a receiver operating characteristic (ROC) analysis was also performed to evaluate the models. The ROC curves were constructed by plotting the false positive (1−SP) against the true positive rate (SE) computed and the area under a ROC curve (AUC) revealed whether a model constructed was a random model with an area of 0.5 or an ideal one with an area of 1.0 computed.

## Results and Discussion

### Condensed Fukui function

**Fig 3:**
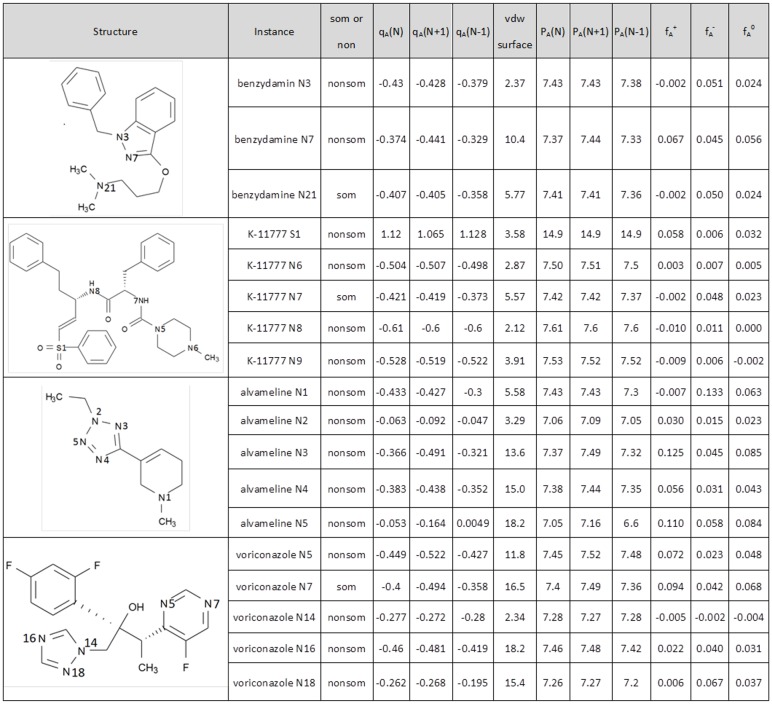
The values of condensed Fukui functions computed for the four selected examples in the training set. q_A_(N), q_A_(N+1) and q_A_(N-1) represent respectively the atomic charge in the molecule with N electrons, the atomic charge in the molecule with N+1 electrons, and the atomic charge in the molecule with N-1 electrons. P_A_(N) is equal to the atomic number of atom A—q_A_(N) and so on. f_A_^+^, f_A_^−^ and f_A_^0^ are the values of condensed Fukui function computed. The values of f_A_^+^, f_A_^−^ and f_A_^0^ represent the electrophilicity of atom A, nucleophilicity of atom A, and radical attack susceptibility of atom A, respectively.

The total number of compounds studied was 85 and which were divided into a training and test set with each carrying roughly the same number of potential SOMs (Table 1). Both the singlet and triplet spin types of each optimized structure were considered in the calculation. The populations P_A_(N), P_A_(N+1), and P_A_(N-1) computed from Mulliken charges via DFT method were used to calculate the condensed Fukui Function. The values of f_A_^+^, f_A_^–^, and f_A_^0^ computed represent the electrophilicity of atom A, nucleophilicity of atom A, and radical attack susceptibility of atom A, respectively. The FMO enzymes are capable of metabolizing the xenobiotics carrying nucleophilic heteroatoms such as nitrogen and sulfur so that the corresponding f_A_^−^ computed could be used to predict SOMs straightforwardly. In Fig 3, we present the values of condensed Fukui Function computed for 4 out of 85 compounds in the dataset. Among these 4 substrates, 3 carrying some N-oxidation SOMs and the last one carrying no SOM since it is a non-substrate of FMO enzymes (Fig 3).

The first drug shown in Fig 3 is benzydamine which is a nonsteroidal anti-inflammatory drug and usually used for pain relief and anti-inflammatory treatment of mouth and throat. There were three nitrogen atoms N3, N7, and N21 on benzydamine being predicted to be three potential SOMs and the corresponding f_A_^−^ computed were 0.051, 0.045, and 0.050, respectively (Fig 3). However, N21 was the actual SOM of the drug though both its f_A_^−^ and charge were not among the highest computed for the three potential SOMs. The actual SOM did not occur at the atom with the highest f_A_^−^ computed which could be due to the substructure preference by FMO enzymes.

There were five potential SOMs including both sulfur and nitrogen atoms S1, N6, N7, N8, and N9 on drug K11777 or k777 (N-methyl-piperazine-Phe-homoPhe-vinylsulfone-phenyl) which is an irreversible cysteine protease inhibitor (Fig 3). The corresponding f_A_^−^ computed were 0.006, 0.007, 0.048, 0.011 and 0.006, respectively (Fig 3). The actual SOM of K11777 was on N7, a tertiary amine atom which also had the highest f_A_^−^ computed among all the potential SOMs identified. However, the charge on N7 was among the second highest as opposite to the highest computed on S1 located in substructure R_2_SO_2_.

Voriconazole is a triazole antifungal drug usually used to treat the serious and invasive fungal infections. Apparently, voriconazole had five potential SOMs and they were on N5, N7, N14, N16 and N18, respectively (Fig 3). While N5 and N7 were located in substructure pyrimidine, a six-members ring; N14, N16, and N18 were located in substructure triazole which was a five-members ring. The f_A_^−^ values of N5, N7, N14, N16 and N18 computed were 0.023, 0.042, -0.002, 0.040 and 0.067, respectively. Note that the actual SOM in voriconazole occurred on N7 and its corresponding f_A_^−^ was the second whereas its charge was the third highest computed among the five potential SOMs identified.

The fourth drug listed in Fig 3 was alvameline which was a M1 receptor agonist and M2/M3 receptor antagonist still under investigation for treating the Alzheimer's disease. The drug was a non-substrate of FMO enzymes and research attempt for the drug was currently ceased due to its poor clinical outcome. Although the drug was a non-substrate of FMO enzymes and no actual SOMs was given, there were five potential SOMs could be seen on N1, N2, N3, N4 and N5 (Fig 3). While N2, N3, N4 and N5 were in substructure tetrazole, a five-members ring, N1 was belonging to the tertiary amine of a six-members ring. The corresponding f_A_^−^ computed for N1, N2, N3, N4 and N5 were 0.133, 0.015, 0.045, 0.031 and 0.058, respectively (Fig 3).

In general, the values of f_A_^−^ computed representing the nucleophilicity of atom A in a drug molecule. We have found that the intramolecular ranking of f_A_^−^ computed was highly correlated with the actual SOMs identified for each drug molecule. All these actual SOMs identified were either having the first or second ranked f_A_^−^ computed. For examples, the f_A_^−^ computed for the actual SOM of K11777 was ranked the highest, while those computed for benzydamine and voriconazole were both ranked the second highest among all the potential SOMs (Fig 3). This would indicate that all these identified actual SOMs had higher nucleophilicity for being recognizable by FMO enzymes. However, as shown in Fig 3, the intramolecular ranking of charge was not as effective as the aforementioned ranking of f_A_^−^ in reflecting the actual SOMs identified. For examples, though the charges for the actual SOMs identified for benzydamine and K11777 were both secondly but not first ranked (Fig 3). In general, more negative charge would mean more nucleophilicity computed. However, unlike f_A_^−^ computed, the charges computed were less effective or could not be used to correctly predict the actual SOMs. Here, both substrates and non-substrates of FMO enzymes were recruited into the training set for building a SVM model. We have found that some higher f_A_^−^ values were computed within the non-substrate molecules. This would imply that simply using the condensed Fukui function computed was insufficient to predict whether a drug could be a substrate or non-substrate of FMO enzymes.

### Attributes from circular fingerprints

**Fig 4:**
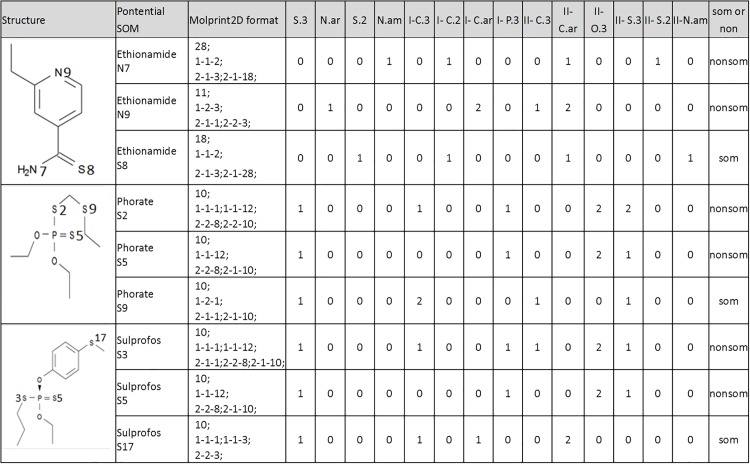
Converting the original Molprint2D text format into numerical values. The original Molprint2D text format are converted to numerical values so that they are readable by the WEKA package. Such a converting for three selected training set compounds are shown in the figure. Label "I-" represents the neighbor type of the first layer while label "II-" represents the neighbor type of the second layer.

We employed the circular fingerprints of Molprint2D [105] to account the influence of surrounding atoms on a particular potential SOM. The format of Molprint2D generated using the 53 SYBYL atomic types C.3, C.2, C.ar, C.1,…, Mo, Mn, and Co.oh was converted into some 53×3 numerical attributes where the first, second, and third column was used to describe the center, first-layer and second-layer neighbors, respectively. For an example, a Molprint2D of 1; 1-1-2; 2-1-9; could be transformed into the first column represented by 53 attributes as 1 0 0 0 …… 0, the second column represented by 53 attributes as 0 1 0 0…… 0, and the third column represented by 53 attributes as 0 0 0 0 0 0 0 0 1 0 0 0…… 0. Fig 4 gave the conversion of Molprint2D fingerprints generated into some numerical attributes for three selected drugs. Note that the columns with all zero values assigned for the hydrogen, sodium,…, and dummy atoms were removed.

### Features and model selection

The initial features employed were consisted by two parts: (1) 10 quantum mechanics features including charges, surface area, and condensed Fukui function computed; and (2) 53 × 3 attributes from layer 0 (central atom) to layer 2 created.

In the quantum features, q_A_(N+1), q_A_(N-1), P_A_(N), P_A_(N+1) and P_A_(N-1) were computed as condensed Fukui functions and were removed for further model training. Because some SYBYL atomic types were excluded from our dataset, the actual number of attributes used was 37 from layer 0 (central atom) to layer 2. Thus, the initial number of features created before feature selection were 42 (5 quantum mechanics features plus 37 attributes from circular fingerprints).

### Performance measurement

We used the grid search provided in the WEKA package to obtain the optimized parameters. The optimized SVM parameters *C*, γ and *W* (weight) after the training process were 15, 1/instances (default), and 1 (default), respectively. Normalization and the probability estimates were used and the other parameters employed were default settings. As shown in Table 3, the non-SOM set was designated as the class I while the SOM set was designated as the class II set. The performance of our training model was measured by sensitivity (SE), specificity (SP), accuracy (ACC), and Matthews correlation coefficient (MCC) computed which were 0.851, 0.864, 0.856, and 0.705, respectively (Table 3).

**Table 3:** The performance of the training and the test set given by the model built.

Method		instances	Features	Parameters			SE	SP	ACC	MCC	AUC
SVM	Training set	111	5+37	C 15	γ 1/111	W 1	0.851	0.864	0.856	0.705	0.887
	Test set	117	5+37	-	-	-	0.790	0.917	0.829	0.659	0.877
Naive Bayes	Training set	111	5+37	Default setting			0.821	0.864	0.838	0.673	0.869
	Test set	117	5+37	Default setting			0.790	0.889	0.821	0.635	0.867
Random Forest	Training set	111	5+37	Default setting			0.910	0.864	0.892	0.774	0.927
	Test set	117	5+37	Default setting			0.840	0.861	0.846	0.668	0.868

In the model training process, class I set was designated as the non-SOM while class II set was designated as the SOM set. Instances represent the number of potential SOMs identified. There were 5 quantum features and 37 attributes from circular fingerprints used for building the model. Parameters C, γ and *W* were obtained from the SVM training. The sensitivity (SE), specificity (SP), accuracy (ACC), Matthews correlation coefficient (MCC), and area under the ROC curve (AUC) were used to characterize the performance of the SVM model built. Symbol "-" was used to represent the same numbers used in the test set.

**Fig 5:**
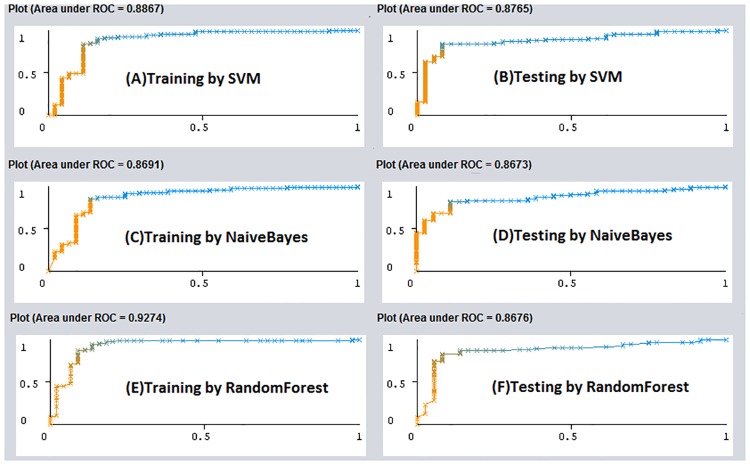
The ROC curves of models. The ROC curves constructed for (A) the training set by the SVM, (B) the test set by the SVM, (C) the training set by the Naive Bayes method, (D) the test set by the Naive Bayes method, (E) the training set by the Random Forest method, and (F) the test set by the Random Forest method.

This would indicate that our model was well-balanced and could accurately differentiate the actual SOMs from the non-SOMs set since both SE and SP computed were high. The Area Under Curve (AUC) of a ROC curve constructed could also reveal the effectiveness of a classification algorithm. A random model would give rise only a point along the diagonal line, while a good model would yield a point in the upper left corner of the ROC curve. Therefore, the AUC for a random and ideal model obtained would be 0.5 and 1.0, respectively. The ROC curve constructed for the test set using parameters obtained from the training set was presented in Fig 5. The AUC of the ROC curve constructed for the test set was 0.887, indicating again the high predicting accuracy of the model constructed. Some other classification methods such as Naive Bayes and Random Forest available from the WEKA [110] package were also employed to build the training models using the aforementioned features. The trained SVM model were also applied to the test set for constructing the ROC curves. As shown in Fig 5, the AUC under the ROC curves obtained from the training by Naive Bayes and Random Forest method with the default settings were respectively 0.869 and 0.927, which would reflect the number of features used was sufficient to represent the datasets. We also employed a test set to test the prediction accuracy of our SVM model constructed. This test set was composed of 117 instances including 40 SOMs and 65 non-SOMs which were collected from some substrates and non-substrates of FMO enzymes. The values of SE, SP, ACC, and MCC computed for the test set were 0.790, 0.917, 0.829, and 0.659, respectively (Table 3), since 33 out of 36 SOMs and 64 out of 81 non-SOMs were correctly predicted. As shown in Fig 5B, the AUC of the ROC curve computed for the test set was 0.877, indicating again both the features used and the SVM model constructed were adequate in predicting the SOMs of FMO enzymes. The prediction models built by both the NaiveBayes and RandomForest methods were also cross-validated using the jackknife (leave-one-out) method. The corresponding AUC computed were 0.8911, 0.8667 and 0.9095 for SVM, NaiveBayes, and RandomForest, respectively (S1 Fig), indicating that all the three models built can accurately predict the SOMs.

**Fig 6:**
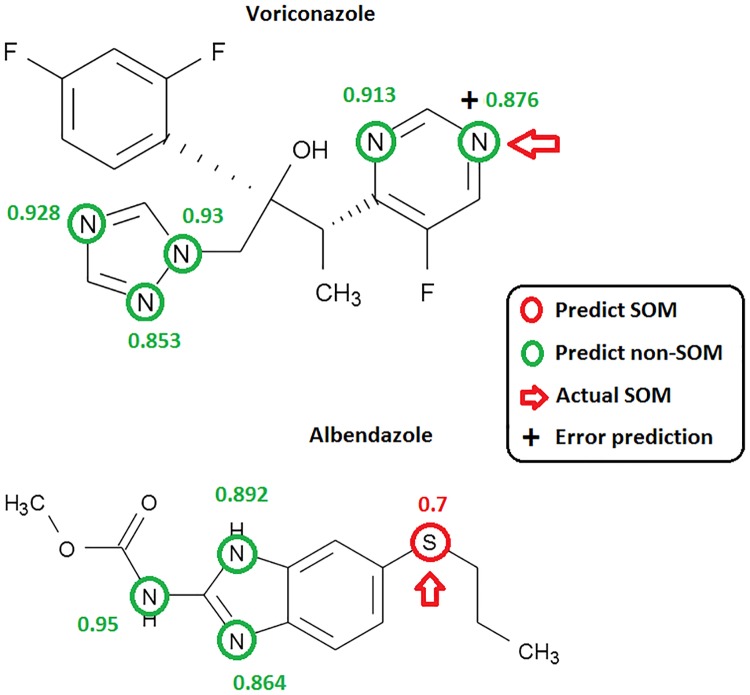
The prediction probability of each potential SOM computed for two selected FMO substrates voriconazole and albendazole. The actual SOMs of each compound determined are highlighted with red arrows. Each predicted SOM is marked with a red circle where the prediction probability computed is shown alongside. Each predicted non-SOM is marked by a green circle and the prediction probability computed is also shown alongside. Symbol "+" is used to denote a false prediction.

As shown in Table 3, lower SE values in the test set obtained might be caused by bulky size or unfitted conformation of the ligands that were expelled from the enzyme active sites. For examples, no activity is observed for the phenothiazine derivative with an alkyl side chain of 3 carbon length (3PTZ in our data set), though Km measured for carbons 5 and 8 derivatives (5PTZ and 8PTZ) were 18 and 10 μM, respectively. The SVM model constructed was unable to lower the prediction error of non-SOM caused by these problems. The other factors which might affect the prediction accuracy of the test set would be the total number of instances used. At present, increasing the data size is still an impractical approach since there are not many human FMO substrates around and those identified for other species such as pig, rabbit, or rat are also scarce as well. The prediction probability of each potential SOM for two selected substrates voriconazole and albendazole was shown in Fig 6, while the total prediction probabilities for the test set were listed in S3 Table.

### Compare with the prediction results given by MetaPrint2D

MetaPrint2D [22] and Metasite [21] are the two known packages that can be also used to predict SOMs by Phase I enzymes. The former was a free online tool and was employed here on the same test set for comparing with our prediction results by the SVM models built. MetaPrint2D encodes each SOM and its corresponding substructure with circular fingerprints. Then, it screens a specific metabolic reaction and calculates the occurrence ratio (likelihood) of each SOM involved in the metabolic reaction. The number of exact levels and similarity threshold chosen were 3.0 and 0.5 which were both default settings. The weights of the six fingerprint levels used were 1.0, 1.0, 1.0, 0.75, 0.5, and 0.25, respectively. On output, each atom in a molecule was color coded by its normalized occurrence ratio (NOR) computed. Higher NOR computed means higher reported rate of being a metabolism site in the database. An atom was colored in red, orange, green, white, and grey to represent the corresponding NOR computed as 1.00~0.66, 0.66~0.33, 0.33~0.15, 0.15~0.00 and no data, respectively. Note that the substrates of all the phase I metabolic including FMOs enzymes were accounted in the training base by MetaPrint2D. We intended only to compare the true negative rate or SP (Eq 6) computed by MetaPrint2D with our SVM models since those predicted by the former would be much broader than those by the latter. Apparently, there were 22 out of 36 SOMs being correctly predicted by MetaPrint2D as shown in Table 4, If we defined atoms coded with red or orange color as being a potential SOM predicted by the method. However, the corresponding SP computed for the prediction result was 0.611 which was worse than that (0.917) given by our SVM model. This might be ascribed to the fact that fewer human FMO substrates was collected in MetaPrint2D than in our SVM model in the training procedure.

**Table 4:** Comparison between the prediction results for the test set by the SVM model built and Metaprint2D.

	Numbers of instances	Enzyme for SOM	TP	FN	SP
			FP	TN	
Test set	117	FMO	64	17	0.917
			3	33	
Metaprint2D	117	Phase I enzymes	63	18	0.611
			14	22	

Class I set is designated as the non-SOM while class II set is designated as the SOM set in the SVM model built. The number of substrates used in both sets is the same. The performance of the method is characterized by TP (True Positive), FP (False Positive), FN (False Negative), TN (True Negative), and specificity (SP) computed.

## Conclusion

In this report, we have developed a SOM prediction method for some FMO enzymes using SVM with some quantum mechanics and circular fingerprints attributes. The total number of molecular descriptors used in building the SOM prediction model was 42, including 5 quantum mechanics features such as Mulliken charges, condensed FuKui Functions f_A_^+^, f_A_^–^, f_A_^0^, and vdw surface area and 37 circular fingerprints attributes. The prediction ability for SOM by the SVM model constructed was validated by both the training and the test sets and was found to be accurate. We have also compared the prediction result on the test set by our SVM model with that by a free online method MetaPrint2D. Unlike our SVM model which only focusing on FMO enzymes, all the phase I metabolic including FMO enzymes were considered by MetaPrint2D. There are some drugs that are actually not metabolized by CYP450 but rather by FMO enzymes such as benzydamine, itopride, and arbidol. Though FMO are also important phase I metabolic enzymes, it appears that far less attention has been paid to the enzymes than the CYP450 systems. Moreover, a major difference between FMO and CYP450 is that the adverse drug-drug interaction problem may be avoided for drugs that are solely metabolized by the former [7, 8]. Though the SVM prediction models constructed by us for only FMO enzymes may deserve merit, there are still rooms for improvement for our current models constructed especially in generating a correct bound conformation for a substrate. The current SVM methodology may be also extended for constructing the prediction models for the CYP450 substrates only.

## Supporting Information

S1 Fig The ROC curves of all the SVM, NaiveBayes, and RandomForest models built and cross-validated by the jackknife (leave-one-out) method are given. (TIFF)

S1 Table All the structures, actual SOMs and related references used in the dataset (the diagram of structures are obtained from open babel and NCBI) are listed in the table. (PDF)

S2 Table The original data set includes both the training and the test sets are given in the table. (XLS)

S3 Table The prediction probabilities computed for the test set are listed in the table. (XLSX)
