# Supplementary figures and images for: Predicting the Metabolic Sites by Flavin-Containing Monooxygenase on Drug Molecules Using SVM Classification on Computed Quantum Mechanics and Circular Fingerprints Molecular Descriptors
Source: PLoS One. 2017 Jan 10;12(1):e0169910. doi: 10.1371/journal.pone.0169910 (PMC5224990; doi:10.1371/journal.pone.0169910)

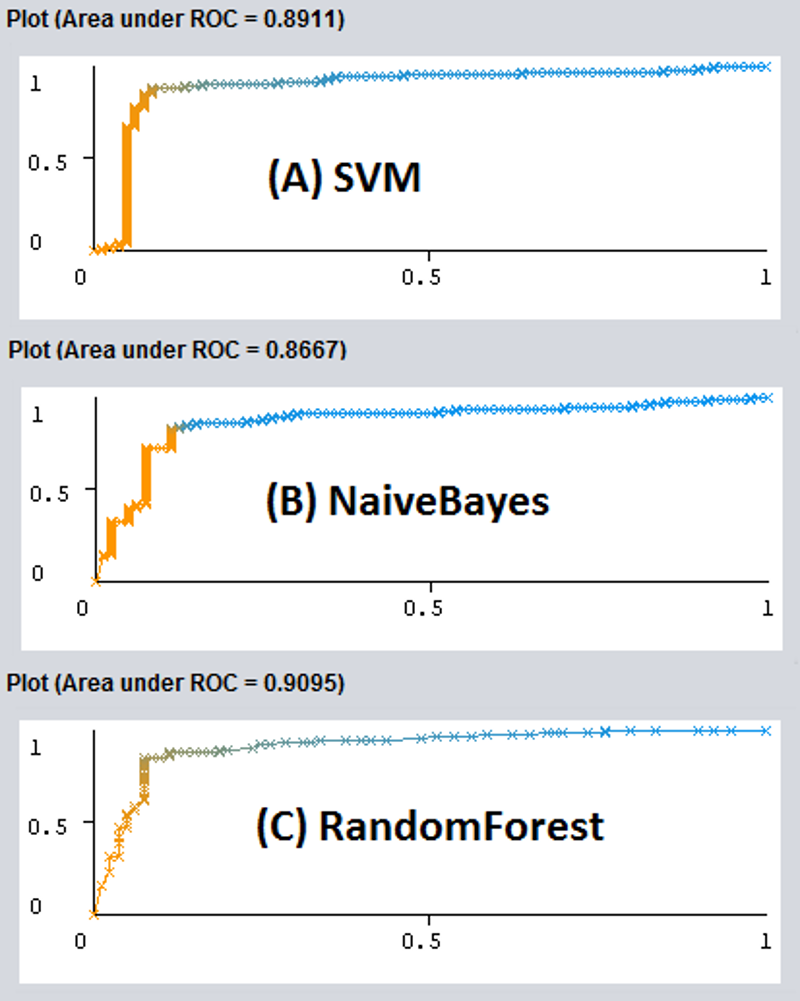

Supplement: S1 Fig — (TIFF) [file pone.0169910.s001.tiff]
