# Supplementary material for: Predicting the Metabolic Sites by Flavin-Containing Monooxygenase on Drug Molecules Using SVM Classification on Computed Quantum Mechanics and Circular Fingerprints Molecular Descriptors
Source: PLoS One. 2017 Jan 10;12(1):e0169910. doi: 10.1371/journal.pone.0169910 (PMC5224990; doi:10.1371/journal.pone.0169910)
Supplement: S1 Table — (PDF) [file pone.0169910.s002.pdf]

Compound	FMO	Product	Reference
Cysteamine	hFMO2	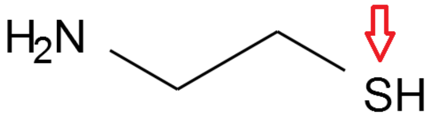	13
Methionine	hFMO3	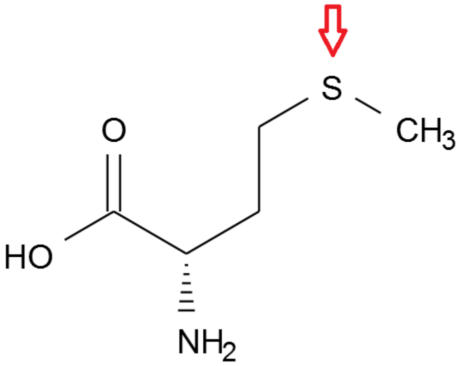	41
Trimethylamine	hFMO3	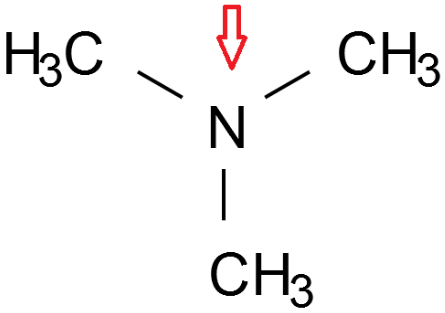	42
Amphetamine	hFMO3	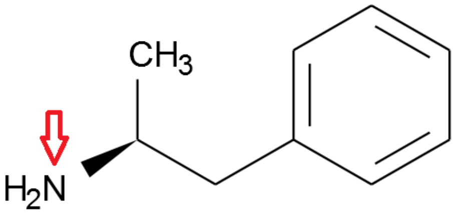	43

Phenethylamine	hFMO3	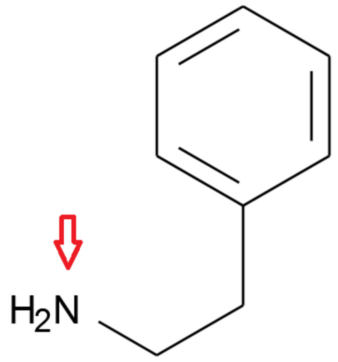	44<br>45
Tyramine	hFMO3	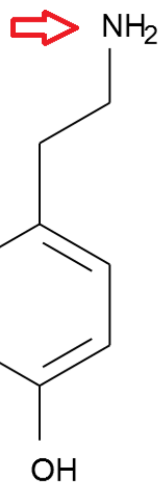	46
N-deacetyl ketoconazole	hFMO1	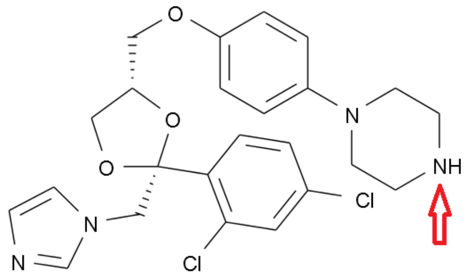	47
Methamphetamine	hFMO3	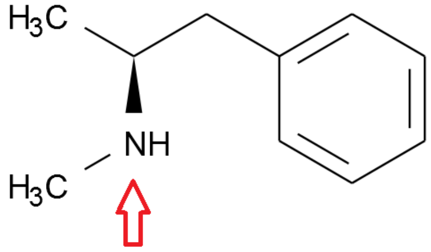	43

ABT-418	hFMO3	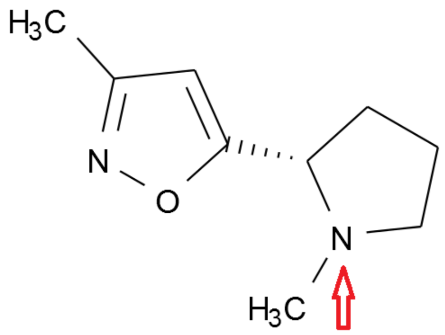	48
Benzydamine	hFMO1<br>hFMO3	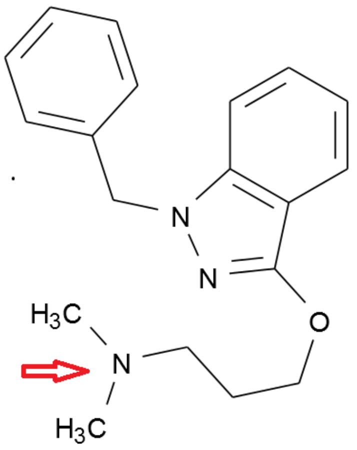	49
Clozapine	hFMO3	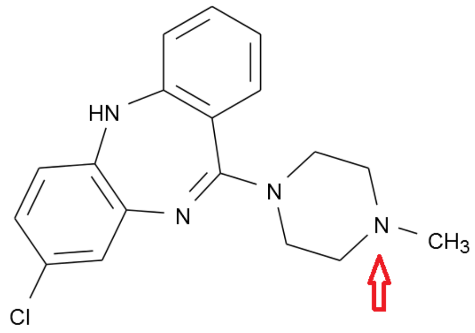	50
Itopride	hFMO1 hFMO3	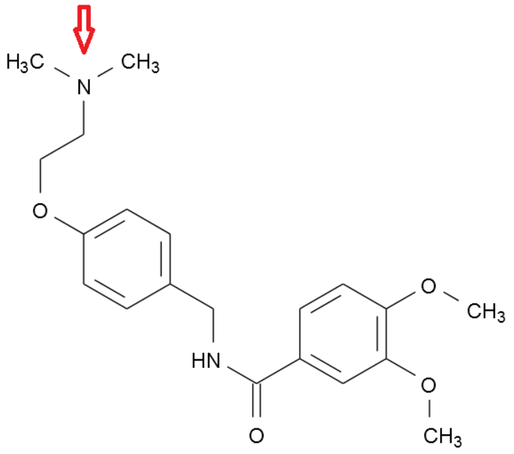	51

K11777	hFMO3	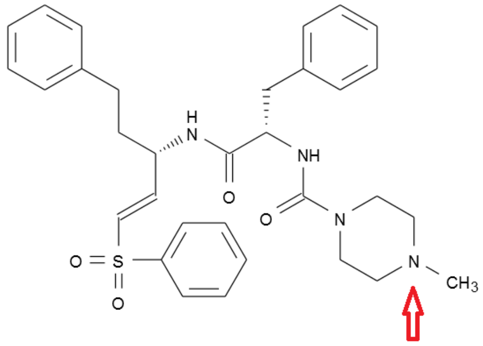	52
(S)-nicotine	hFMO3	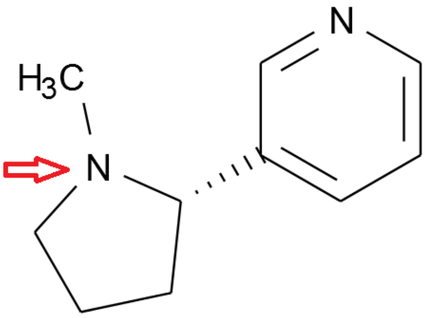	53
Olopatadine	hFMO1 hFMO3	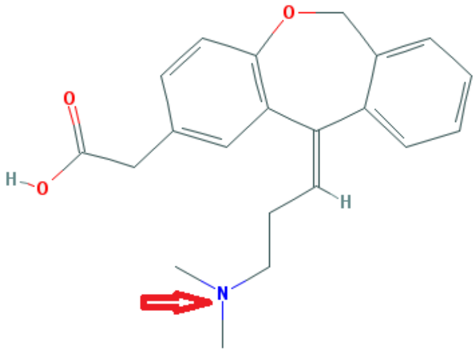	54
S 16020	hFMO3	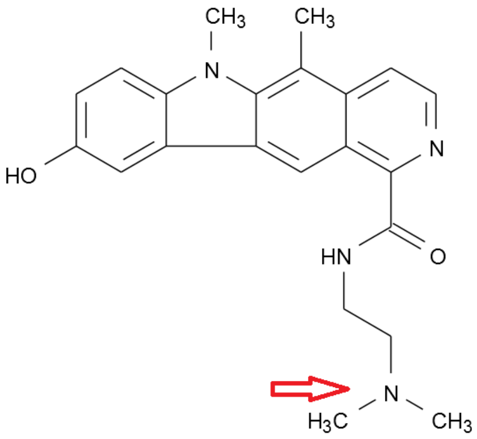	79

SnI-2011 (Cevimeline)	hFMO1	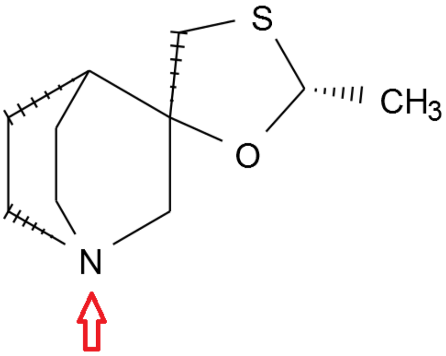	55
Tamoxifen	hFMO3	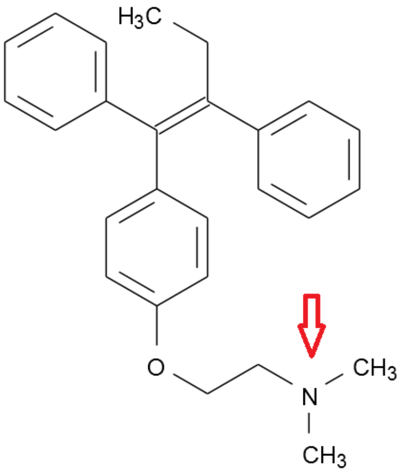	13
Xanomeline	hFMO3	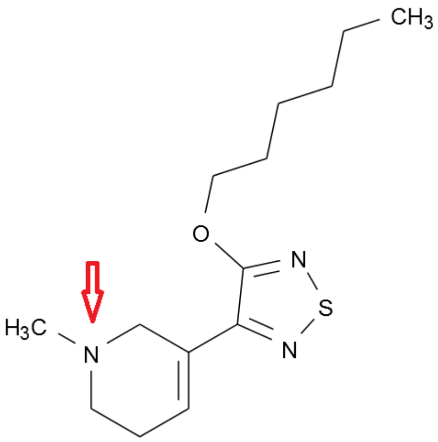	56

Disulfoton	hFMO1<br>hFMO2	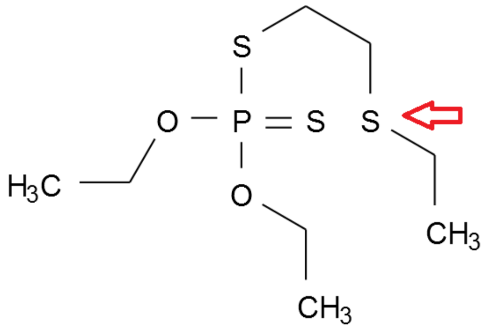	57
Methiocarb	hFMO1	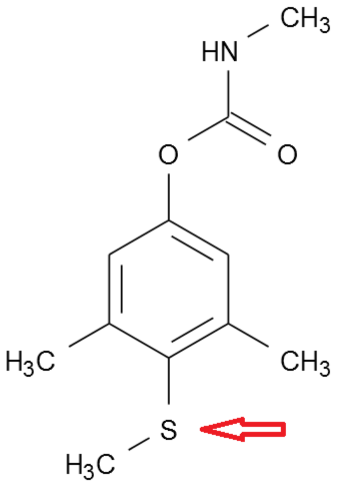	58
S-methyl-N-N-dithiocarbamate	hFMO1	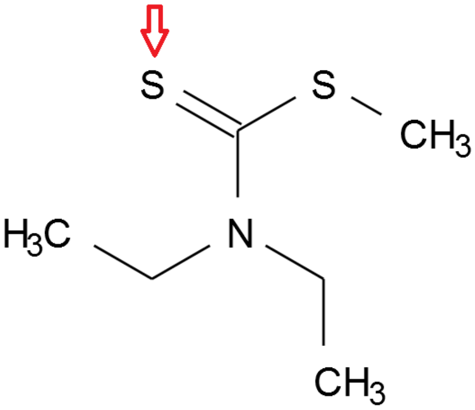	59

S-methyl-esonarimod (KE-748)	hFMO1,3,5	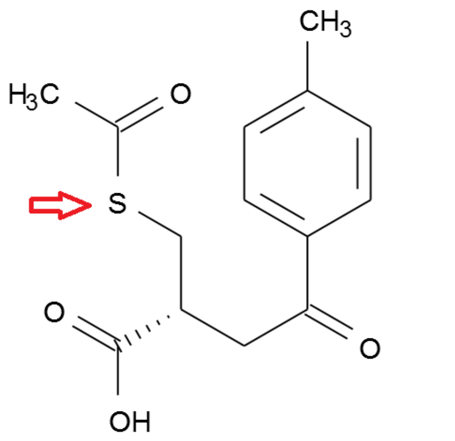	60
M25	hFMO1,3,5	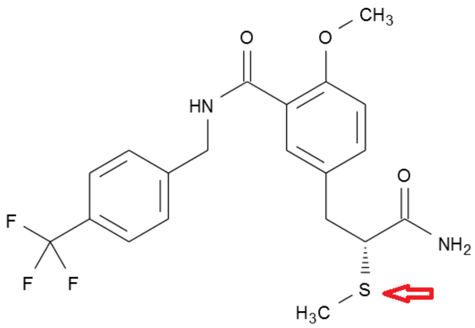	61
M25(dl form)	hFMO1,3,5	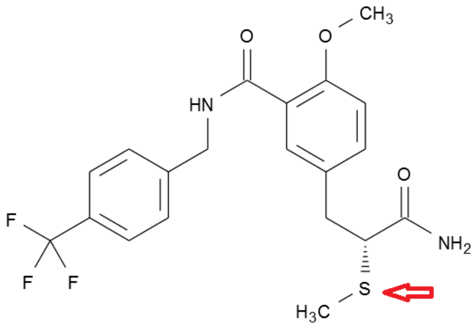	61
Phorate	hFMO1<br>hFMO2	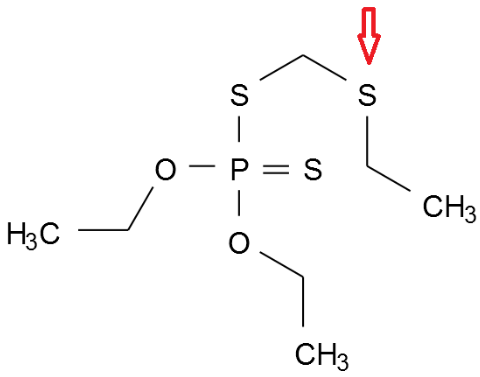	80

Sulindac sulfide	hFMO3	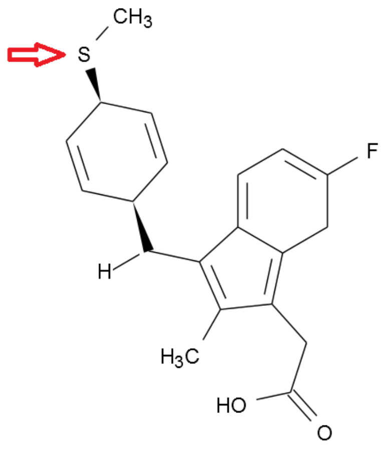	62
Sulprofos	hFMO1	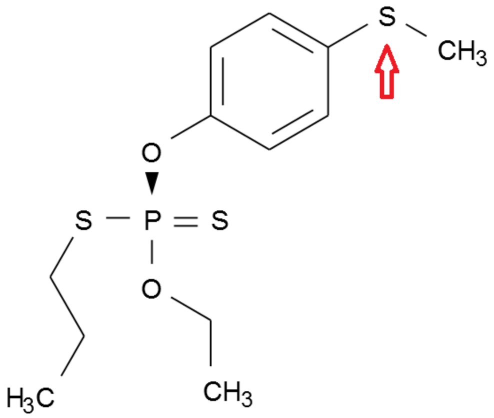	80
Tazarotenic acid	hFMO1,3	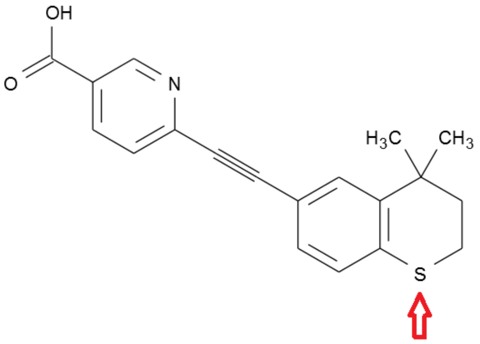	63
Butyl p-tolyl sulfide	hFMO3	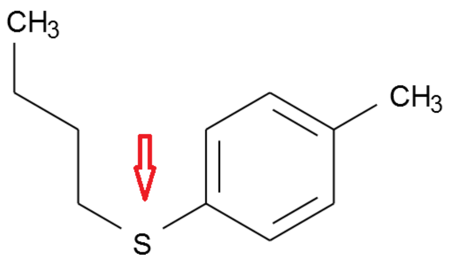	64

Methyl p-tolyl sulfide	hFMO3	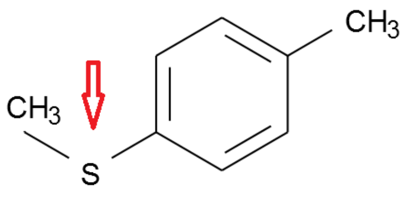	64
Ethionamide	hFMO2<br>hFMO3	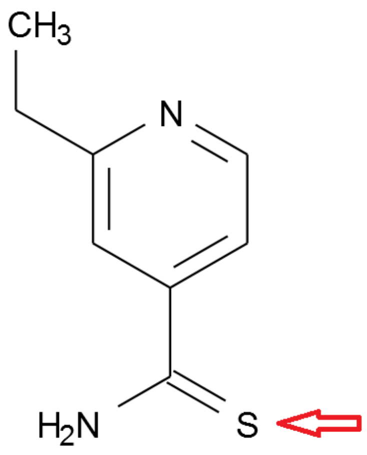	13
Ethylene thiourea	hFMO2	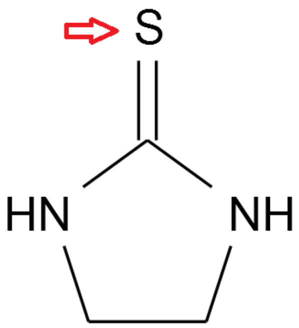	65
α-Naphthylthiourea<br>(1-Naphthylthiourea)	hFMO2	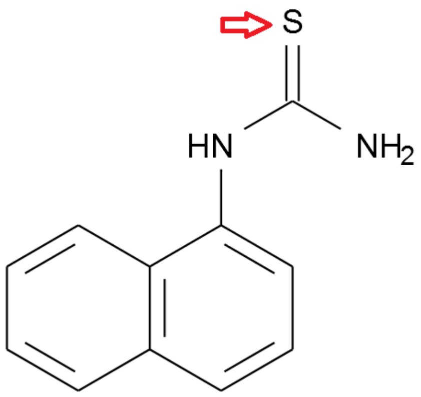	66

1-Phenylthiourea	hFMO1<br>hFMO2	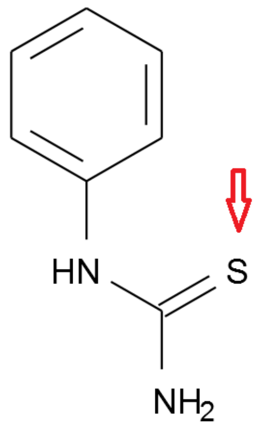	66
Thioacetanilide	hFMO2	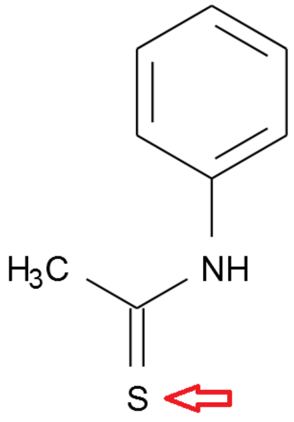	65
Thiobenzamide	hFMO2	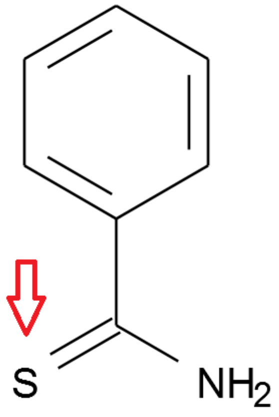	65

Thiourea	hFMO1<br>hFMO2	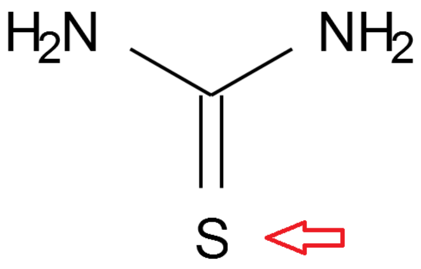	67
diphenhydramine	hFMO	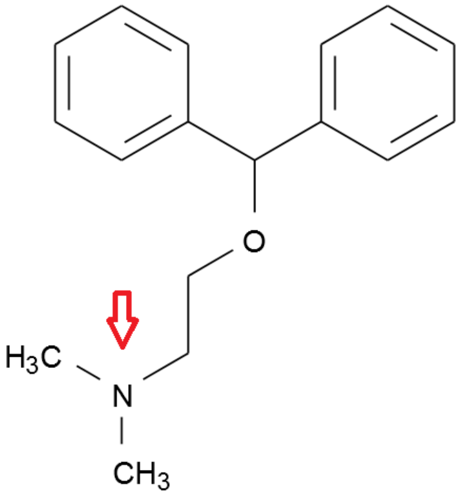	21
AH-262_36084007	hFMO	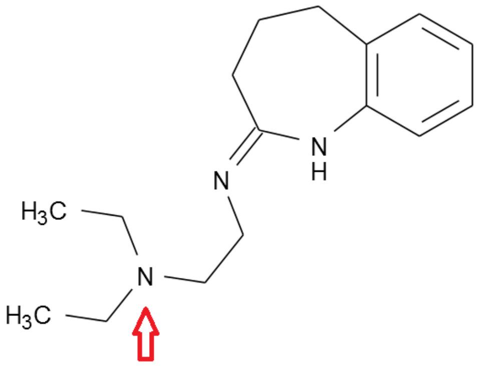	21

Alvameline	No reaction	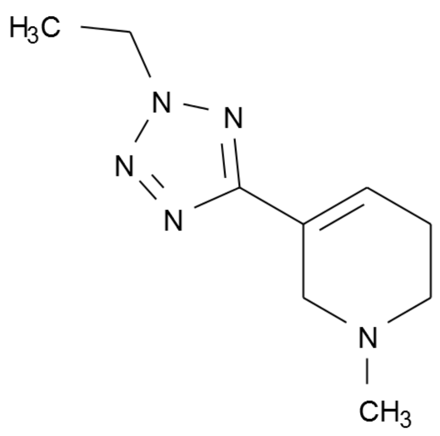	21
AN-465_42886206	hFMO	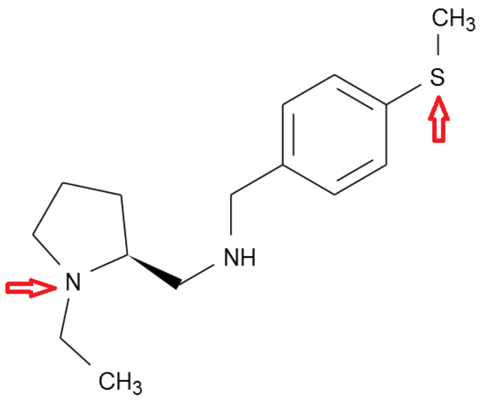	21
AN-465_42886238	hFMO	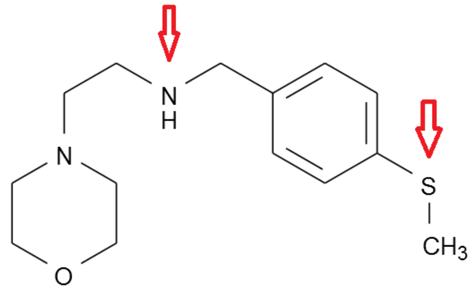	21
AN-465_42886418	hFMO	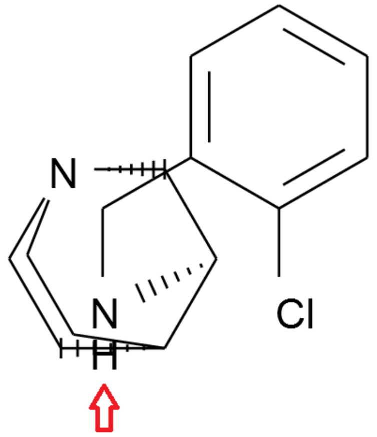	21

AN-465_42886511	hFMO	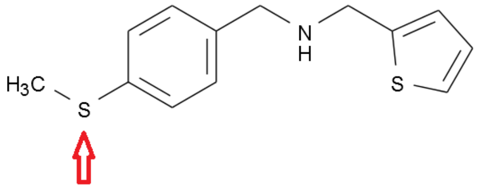	21
AN-465_42889117	hFMO	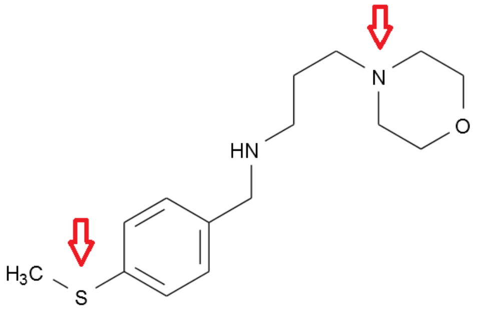	21
AN-465_43384061	hFMO	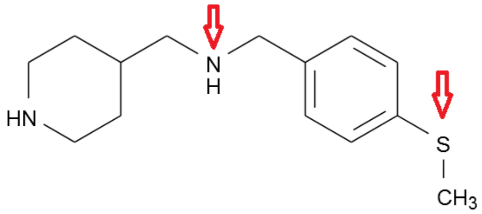	21
Arecoline	hFMO	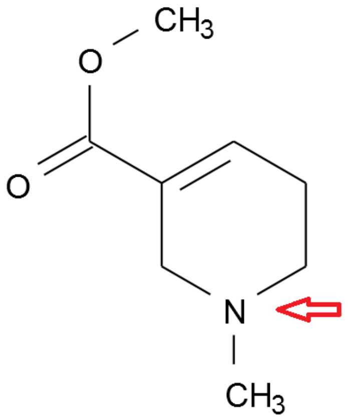	21

Bepridil	No reaction	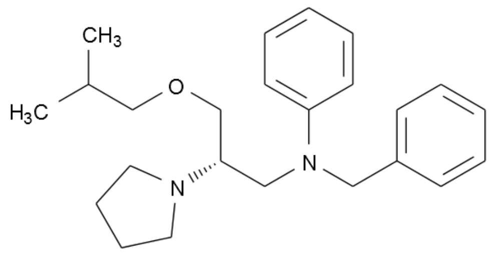	21
BPEA	hFMO	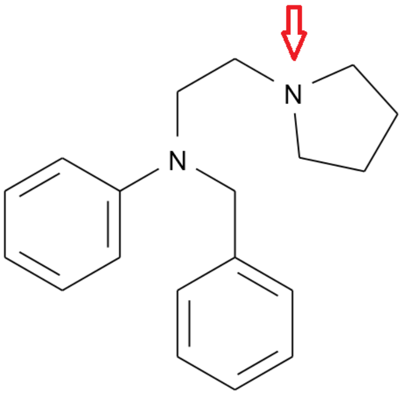	21
Cimetidine	hFMO	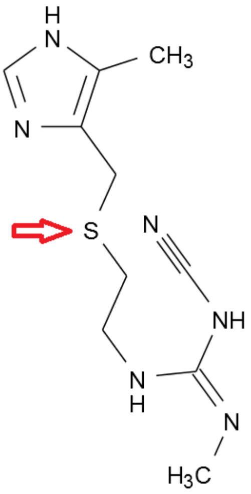	21
cyamemazine	No reaction	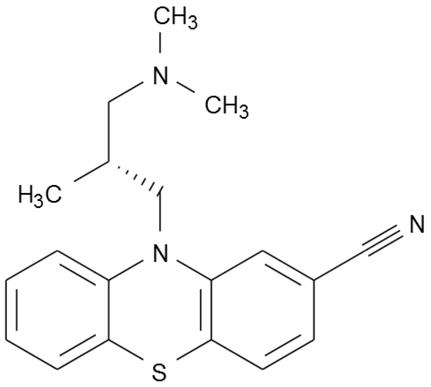	21

Dapsone	hFMO1<br>hFMO3	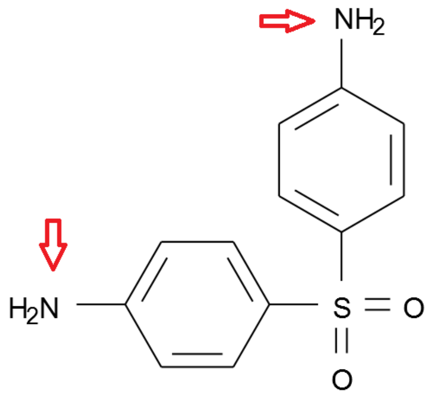	81
sulfamethoxazole	hFMO1<br>hFMO3	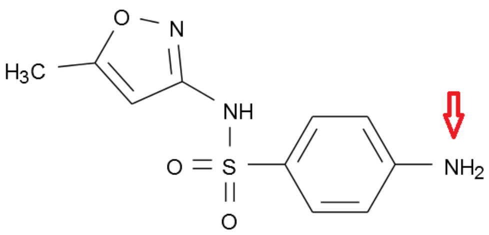	81
Tetrahydroisoquinoline	hFMO<br>(but very weak substrate)	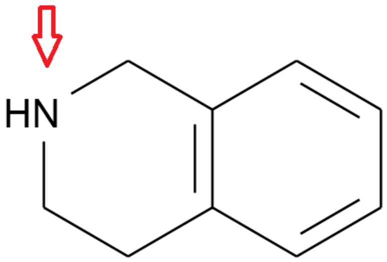	21
Voriconazole	hFMO3	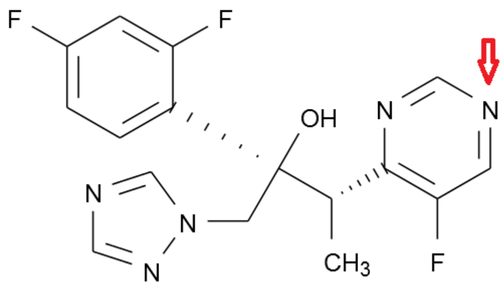	82

Zotepine	hFMO	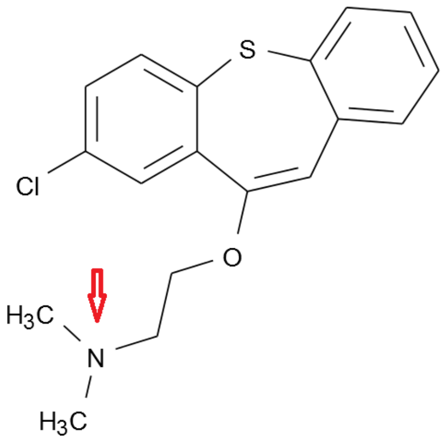	21
Imipramine	hFMO	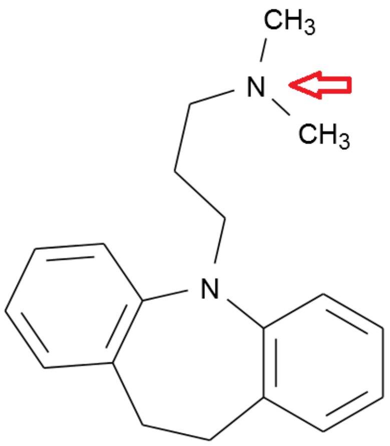	21
3PTZ	No reaction	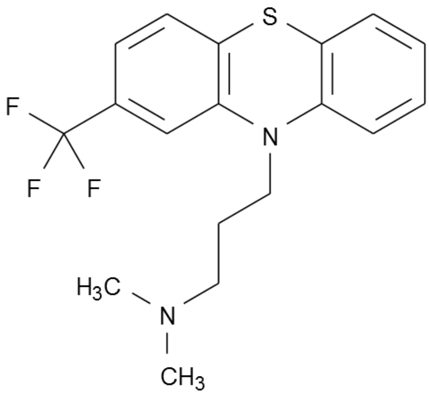	13

5PTZ	hFMO	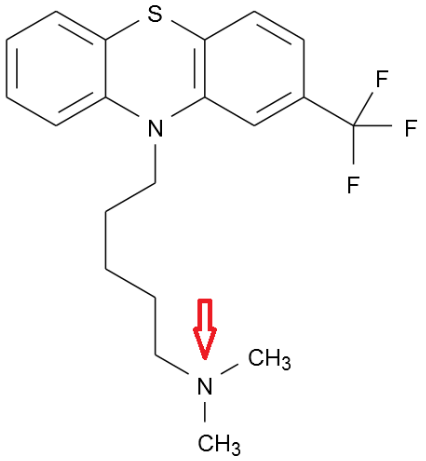	13
8PTZ	hFMO	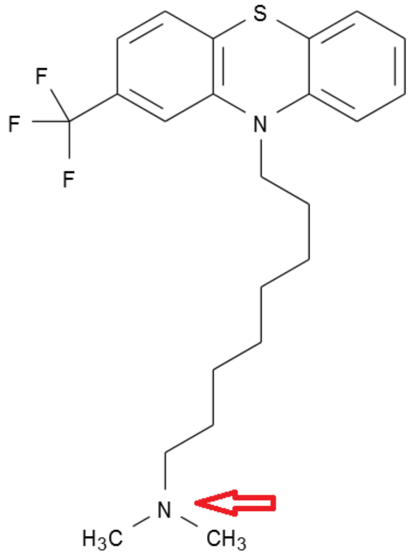	13
Caffeine	hFMO	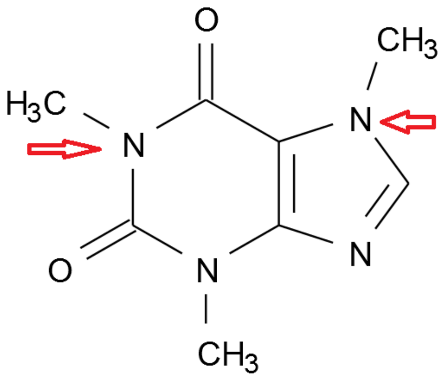	69

Demeton_o	hFMO	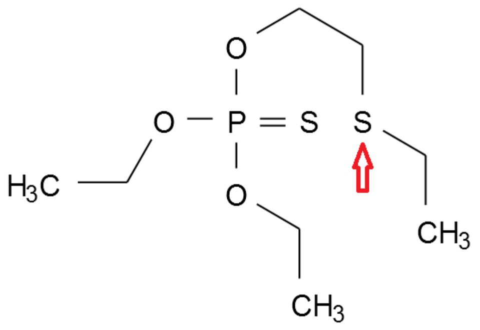	58
carbophenothion	No reaction	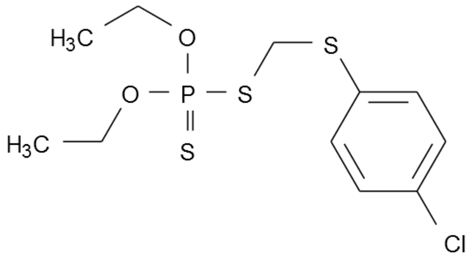	58
Ethiofencarb	hFMO	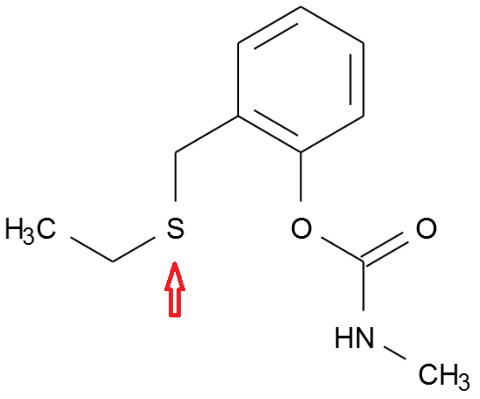	58
Fenthion	hFMO	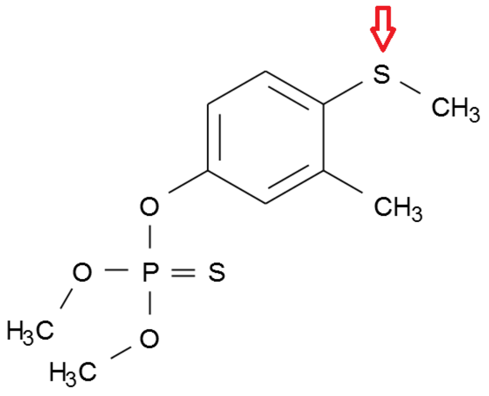	58

Fonofos	hFMO	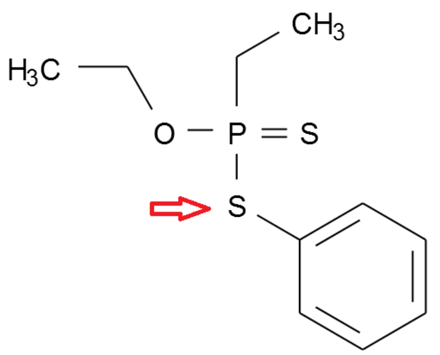	58
Chlorpromazine	hFMO	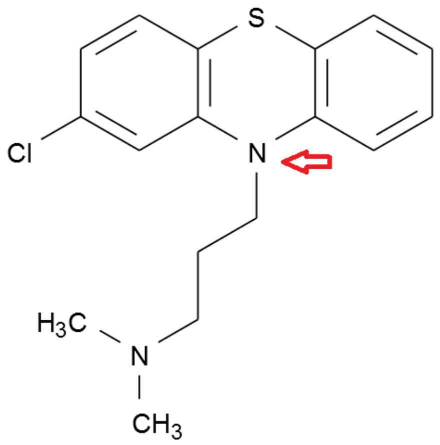	67
Ranitidine	hFMO	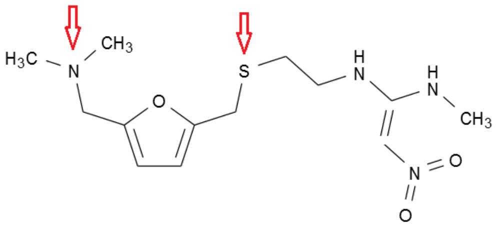	71
Darexaban	hFMO	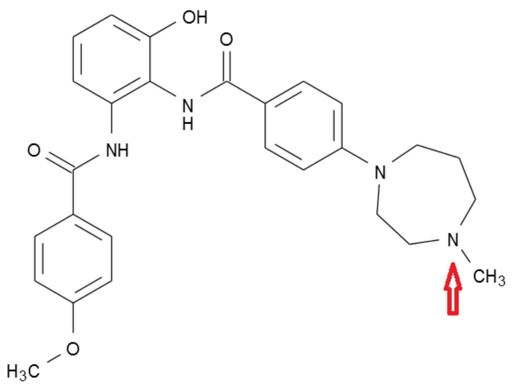	72

moclobemide	hFMO	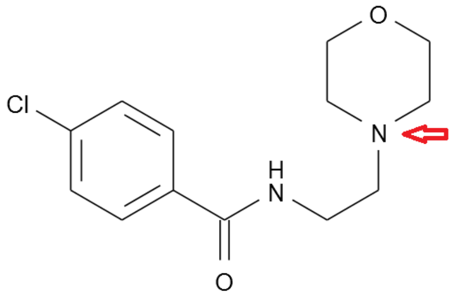	73
MPTP	hFMO	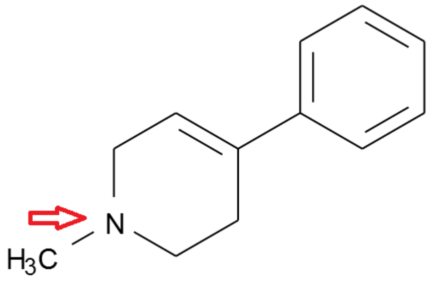	74
S-benzyl-L-cysteine	hFMO	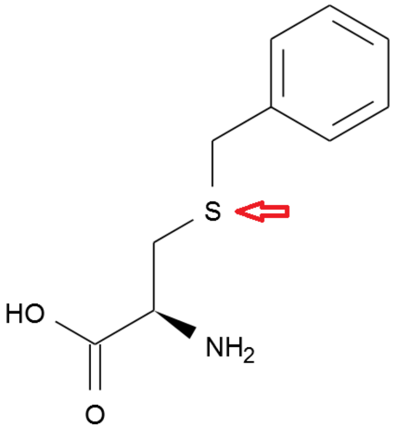	13
Methimazole	hFMO	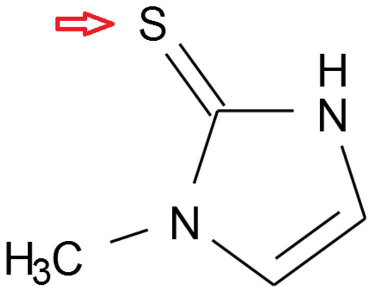	76

Tozasertib	hFMO	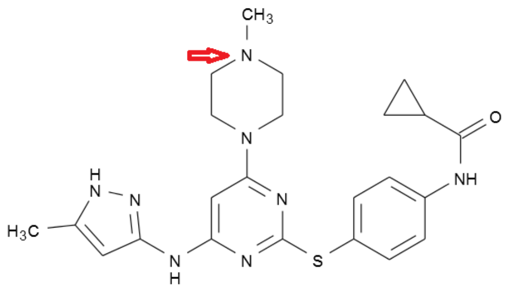	76
Danusertib	hFMO	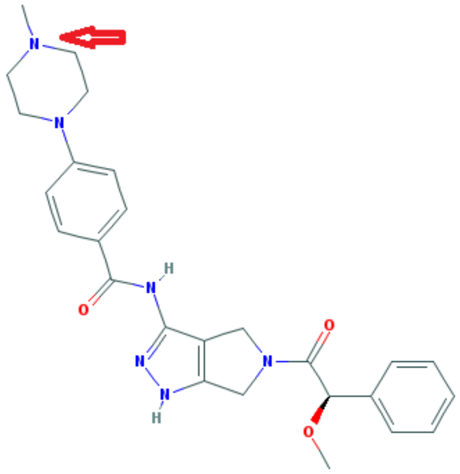	77
N-Dodecylhydroxyamine	No reaction	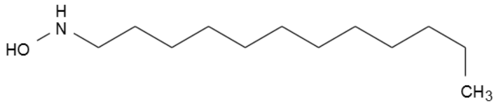	65
Cocaine	No reaction	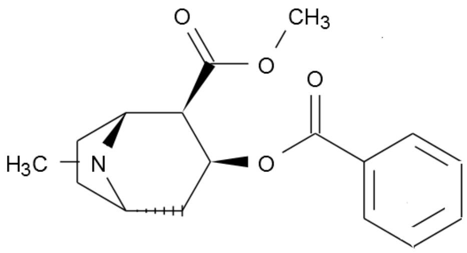	65

Phencylidine	No reaction	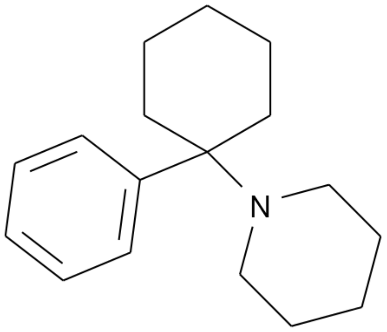	65
Deprenyl	No reaction	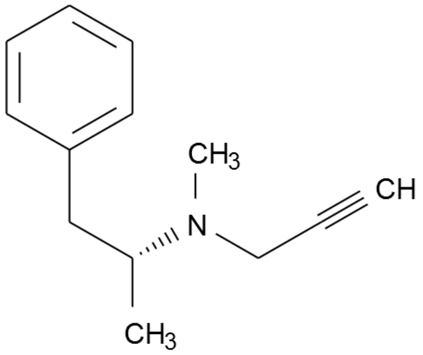	65
1,3-Diphenylthiourea	No reaction	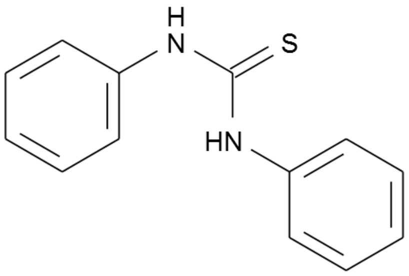	67
AGN9	No reaction	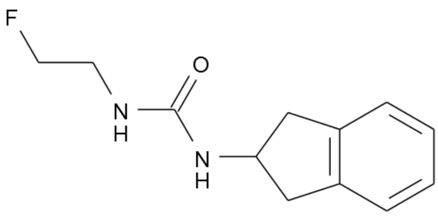	78

AGN10	No reaction	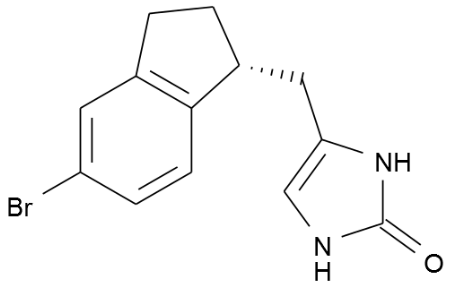	78
AGN11	FMO1<br>FMO2	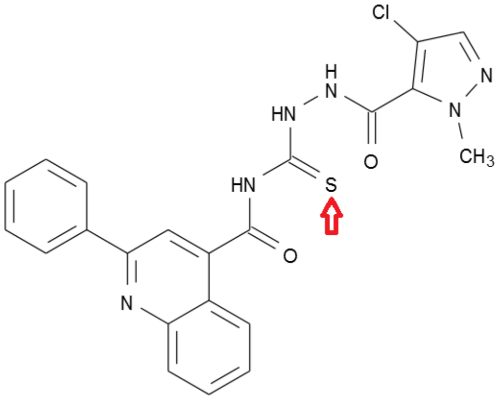	78
Albendazole	hFMO	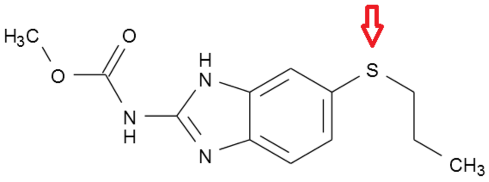	13
The substrates below are not yet included in the dataset for model.			
Arbidol	FMO1<br>FMO3<br>FMO5	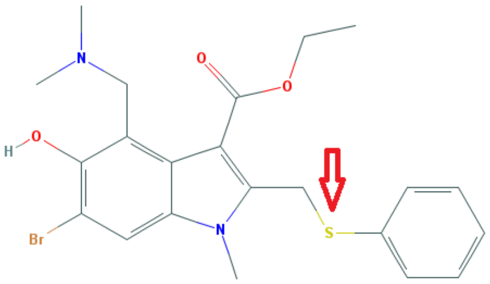	11

Olanzapine	FMO3	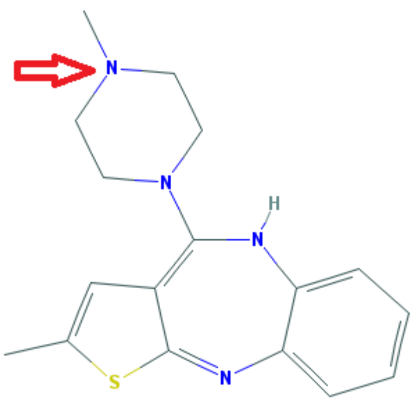	83.
TG100435	FMO	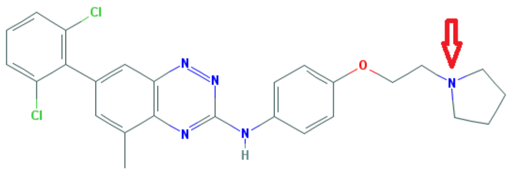	15
N,N-dimethylamphetamine	FMOs	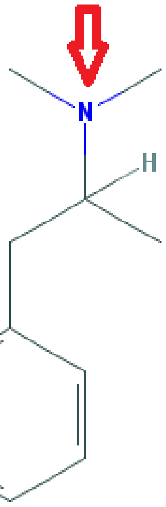	84
Almotriptan	FMO3	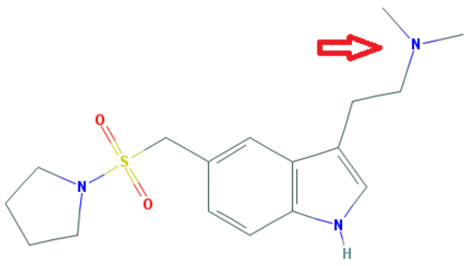	85

Dasatinib	FMO3	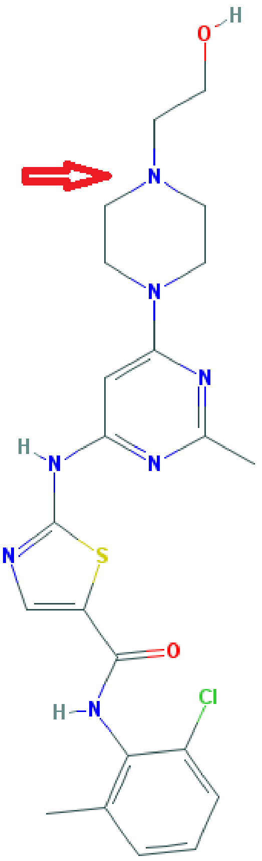	86
ziprasidone	hFMO (NOX but SOM unsure)	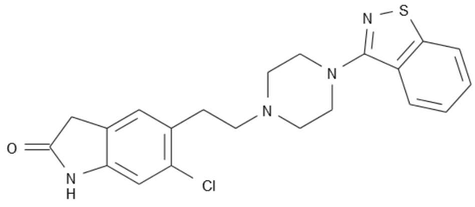	21
